# Response to the Letter regarding “The role of reviewers in the era of systematic reviews and meta-analysis: A practical guide for researchers”

**DOI:** 10.17305/bb.2026.14271

**Published:** 2026-04-13

**Authors:** Emir Begagić, Faruk Skenderi, Semir Vranić

**Affiliations:** 1 Department of Neurosurgery, Cantonal Hospital Zenica, Zenica, Bosnia and Herzegovina; 2 Biomolecules and Biomedicine, Sarajevo, Bosnia and Herzegovina; 3 College of Medicine, QU Health, Qatar University, Doha, Qatar

We express our gratitude to Dr. Mondal for his insightful and constructive correspondence [1] regarding our article on the review of systematic reviews and meta-analyses (SR/MAs) [2]. His comments effectively extend the dialogue to include several practical pressures that increasingly influence contemporary peer review.

We concur that the primary issue surrounding artificial intelligence (AI) is not the mere application of AI-assisted tools, but the lack of transparent disclosure and rigorous human verification. As previously noted, undisclosed or inadequately supervised AI usage may impact not only the wording but also citation accuracy, interpretive reliability, and the overall trustworthiness of the manuscript [2, 3]. However, limited use of such tools for language enhancement may be acceptable if openly declared and critically evaluated by the authors themselves [2]. Ultimately, accountability for the final scholarly product must remain with humans.

The remarks regarding similarity reports are particularly significant. In evidence syntheses, portions of the Methods section necessarily rely on standardized terminology, established reporting frameworks, and recurrent analytical language. Therefore, similarity scores should not be interpreted in a rigid or decontextualized manner. An indiscriminate reliance on numerical thresholds may promote superficial paraphrasing, reduce clarity, and produce distorted phrasing that undermines scientific communication rather than enhancing it [2]. Moreover, similarity metrics should not be viewed solely in one direction. Our recent preprint on retracted systematic reviews and meta-analyses associated with paper mills indicates that low textual similarity does not preclude inauthentic or manipulated manuscripts and may align with strategies designed to evade plagiarism detection [4]. Thus, similarity assessment should be interpretive rather than mechanical.

We also share the perspective that reference verification has become an increasingly demanding aspect of the reviewer’s role. Inaccurate, fabricated, or poorly matched citations are no longer peripheral issues, particularly in an environment influenced by AI-assisted drafting and industrialized forms of publication misconduct. Our recent preprint revealed that referencing concerns were significantly more prevalent among paper mill-associated retracted SR/MAs than among other retracted evidence syntheses, highlighting that citation checking is now a core research integrity issue rather than a minor editorial detail [4]. This concern is further supported by recent evidence demonstrating that paper mill articles have already tainted systematic reviews in the life sciences [5]. Nevertheless, this burden cannot reasonably rest solely on peer reviewers. Editorial prescreening, automated reference validation, and enhanced publisher-side technical support should shoulder more of this responsibility before manuscripts are submitted for external review.

Dr. Mondal’s observations underscore a crucial point: rigorous peer review of SR/MAs now necessitates more than methodological literacy; it also requires sensitivity to indicators of textual manipulation, unreliable referencing, and emerging forms of misconduct that may manifest with fluency rather than obvious errors [5].

We appreciate this valuable contribution to the discussion and agree that maintaining the credibility of evidence synthesis will depend on a coordinated effort among authors, reviewers, editors, and publishers.

## Data Availability

All data are contained within the manuscript.
